# Occupational Physicians’ Perspectives on Determinants of Employee Participation in a Randomized Controlled Musculoskeletal Health Promotion Measure: A Qualitative Study

**DOI:** 10.3390/ijerph17207445

**Published:** 2020-10-13

**Authors:** Kristina Schubin, Lara Schlomann, Lara Lindert, Holger Pfaff, Kyung-Eun Choi

**Affiliations:** Institute for Medical Sociology, Health Services Research and Rehabilitation Science, Faculty of Human Sciences, Faculty of Medicine, University of Cologne, 50933 Köln, Germany; lara.schlomann@uk-koeln.de (L.S.); lara.lindert@uk-koeln.de (L.L.); holger.pfaff@uk-koeln.de (H.P.); kyung-eun.choi@uk-koeln.de (K.-E.C.)

**Keywords:** occupational physician, workplace health promotion, employee, qualitative research, andersen model

## Abstract

Occupational physicians (OPs) are key figures for advising employees and employers about prevention and health at the workplace. However, knowledge of their views on participation in health promotion measures is sparse. This qualitative study aims to explore occupational physicians’ experiences with employee participation in a randomized controlled workplace measure for musculoskeletal disorders (MSDs) in Germany. We conducted eight semi-structured telephone interviews with occupational physicians. Interviews were transcribed verbatim and analyzed using a combination of conventional and directed content analysis. Findings were mapped based on Andersen’s behavioral model of health services use, resulting in four categories and 10 subcategories. (a) Contextual factors of the measure comprised impacts of the healthcare system and company environment, (b) individual factors of measure participation comprised demographic, social, belief, and MSD need characteristics, (c) health behavior during the measure included OPs’ communication, employees’ personal practices and measure participation, and (d) outcomes of participation included health status, satisfaction, and dissatisfaction with the measure. Findings imply occupational physicians’ and employees’ views should be investigated on a broader scale. Researchers should use present statements for the development of intervention studies, while political and managerial authorities can improve organizational conditions of prevention based on these findings.

## 1. Introduction

### 1.1. Evidence Base and Objectives

As numerous declarations point out, Workplace Health Promotion (WHP) can facilitate health and well-being among a broad population of workers [1,2,3]. Musculoskeletal disorders (MSDs) present a major challenge for WHP. In 2018, MSDs were the most frequent cause of incapacity to work among those insured by statutory health insurance in Germany. They were ranked first among those insured by company health insurance funds with 23.8% of all days of incapacity to work [4]. MSDs describe all diseases, complaints, or injuries of the musculoskeletal system, most often affecting spine and back. MSDs are among the most common causes of chronic pain, physical functional limitations, and a loss of quality of life worldwide [5]. Concurrently, employees seek medical help only when symptoms are present and when there has already been a loss in productivity or working capacity [6]. Research suggests workplace physical activity interventions can promote health and worksite outcomes [7], but intervention acceptability and adherence need further investigation [8].

For WHP, occupational physicians (OPs) take on a special position at the workplace setting in preserving employment [9]. OPs are responsible for advising both employees and employers about occupational care, prevention, and the interplay of work and health. Based on the specific organizational conditions, OPs in Germany contribute to risk assessment, workplace inspections, monitoring, and evaluation of accidents and diseases [10]. On the one hand, their expertise and closeness to employees makes them key informants and referees for WHP and healthcare measures [11,12]. On the other hand, OPs are legally obliged to advise employers about employees’ job rotation and occupational rehabilitation (§ 3 Act on Occupational Physicians, Safety Engineers, and Other Occupational Safety Specialists). Due to their expertise and key role for both individual-related and organization-related aspects, OPs could provide valuable perspectives about phenomena in employees’ WHP participation and improvement thereof. However, it remains unclear how OPs perceive factors of employees’ WHP participation in MSD measures.

Andersen’s model of health services use can be utilized to analyze which individual and contextual factors are relevant for the use of healthcare services [13,14]. The model has been widely used for understanding and structuring results about access to and utilization of health services [15]. Modified versions have been adopted in qualitative studies to assess healthcare utilization from stakeholder perspectives other than actual users, e.g., relatives, caregivers [16,17,18], medical staff, service providers, and key informants [19,20,21]. For instance, a qualitative study similar to the present one investigated medication adherence in patients with rheumatoid arthritis by interviewing rheumatologists, and mapped discovered determinants into Andersen’s model [22]. While awareness of the model has only been developing recently in Germany, it has, for example, been used to investigate predictors of outpatient care utilization [23]. We use the model as a framework for data analysis.

Regarding qualitative evidence about employees’ perspectives on (non-)participation in WHP activities, time and financial constraints were mentioned as major barriers [24]. An explorative, qualitative study aimed specifically at employees’ adherence to physical activity in a workplace setting [25]. It found employees’ low sense of control over factors influencing their intent, lack of incentive and self-efficacy, and negative cost-benefit ratio explained employees’ non-adherent behavior. In a qualitative study on managers’ perceptions of employees’ WHP uptake, further factors on the individual level, the WHP offer itself, and organizational factors were identified [26]. Understanding these factors can be facilitated and complemented by exploring OPs’ perspectives, especially considering the lack of perspectives on organizational factors. Therefore, the present study is guided by the following question: What factors do occupational physicians perceive regarding employees’ participation in a musculoskeletal health promotion measure? More specifically, this study has the objectives of 1. to explore OPs’ perspectives on determinants of employees’ participation in a workplace MSD measure, and 2. to indirectly explore employees’ individual characteristics, behavior, and outcomes of their participation from OPs’ perspectives.

### 1.2. Underlying RCT Study

The present explorative study is embedded within the process evaluation of a randomized controlled trial (RCT) of a musculoskeletal health promotion measure (MHPM). Implemented across 22 German companies in 2017 and tested in a four-year trial, the measure aims to counteract lacking intersectional MSD care. The business sectors comprise of steel and metal manufacturing, automotive industry, technology ventures, trade and service, administrative, and government agencies. The RCT has a multimodal needs-based intervention design focused on physical training. Employees are assigned to one of three modules comprising of early intervention (Module A), rehabilitation (Module B), or reintegration (Module C). Employees who agree to participation are randomly assigned to either assisted self-management (control group) or case management (treatment group) in a module. In the treatment group, case managers offer more work-related diagnostics and support as contact persons to employees. Self-management represents current standard care in Germany. A more detailed description of modules and the MHPM program is provided in Supplementary File 1.

Case managers are the main responsible agents for the coordination of MHPM interfaces, recruitment, and assistance during the RCT. Findings of previous focus groups with these case managers showed randomization proved challenging for recruitment. Perceptions of the intervention’s group superiority, mismatching of participants, necessity of randomization, expectations and reactions of employees, and adapted communication of case managers complicated recruitment [27]. OPs’ experiences with employees’ participation in the RCT were not explored yet. However, OPs play an essential role in the program. They are responsible for taking the medical history of eligible employees, checking their inclusion and exclusion criteria, informing them about modules. and assist recruitment by referring to case managers with a recommendation for a specific module.

## 2. Materials and Methods

A qualitative exploratory study based on an interpretive framework using semi-structured interviews was used [28]. Qualitative investigation, against quantitative investigation, was chosen since OPs’ views on WHP and employees’ measure participation are mostly unexplored. This methodological approach allows for an exploration of a fairly small number of individuals’ experiences. Directed content analysis in combination with conventional content analysis was used for data analysis [29]. Since content analysis is a flexible method to analyze text data, it allowed triangulation of specific content analysis approaches. While data analysis originally began with a general inductive approach, researchers realized during iterative data analysis, that coding patterns resembled elements of Andersen’s model of health services used [13,14]. Following a reflection and discussion of this, the research team decided on combining an inductive approach with a deductive approach, while remaining open for the contents of the data, as analysis “cannot be purely deductive or inductive” [30] (p. 205). Since the lacking state of research about OPs’ views called for a conventional content analysis approach, but the theoretical state of research regarding Andersen’s model allowed a directed approach, these analysis methods were combined to match the research purpose.

Furthermore, semi-structured, individual telephone interviews were chosen to give OPs greater freedom of choosing interview time and place, and to generally lower the administrative threshold for OPs’ participation. Telephone interviews were considered an appropriate method due to the nationwide residence of OPs, as well as the eased reachability and administration of data gathering [31]. Individual semi-structured interviews allowed a more personal, yet systematic interaction setting, and reduce social desirability that e.g., occurs in focus groups. Additionally, telephone interviews further served to avoid recreating a face-to-face consulting situation OPs regularly have with patients, and instead create a more neutral interaction between the OP and researcher [32].

### 2.1. Ethical Approval

The University of Cologne’s Faculty of Medicine’s Ethics Commission reviewed and approved the study (project identification code: 17-171). This study adheres to COREQ guidelines for reporting qualitative research [33].

### 2.2. Accessing the Sample

Purposive sampling was used for the selection of participants. The selection criterium was OPs’ participation in the RCT as referees for the recruitment of employees. Other selection criteria were not applied to maximize OPs’ participation in this study. There was a total of 21 OPs in the trial who were all invited to attend interviews. All OPs were initially approached by e-mail and subsequently contacted by telephone. Eight out of 21 OPs agreed to participate in telephone interviews. Reasons for non-participation were as follows: 3 OPs did not want to participate, and 3 OPs did not consent to audio-recording. Further, 2 OPs were unavailable due to other obligations, 2 OPs were sick during data collection, 2 OPs could not be reached by e-mail or phone, and 1 OP was on parental leave. OPs who were sick and unavailable at the time of initial data collection did not respond to further recruitment attempts. Interviews were conducted individually with 8 OPs in June and July 2018, 12 months after measure implementation.

### 2.3. Setting, Procedure and Data Collection

Data was collected using the audio-recording of telephone interviews. Interviews were conducted by the former research team member LN (sociologist, M.A.), who called OPs from the facilities of the Institute for Medical Sociology, Health Services Research, and Rehabilitation Science. The interviewer left the research team after data gathering and could therefore not be included in the study’s further process. She used a semi-structured, pilot-tested interview guide developed by LL (rehabilitation scientist, M.A.), LS (health economist, M.Sc.), and KEC (psychologist, Ph.D.). All involved researchers were skilled in planning and conducting qualitative research, e.g., through prior academic studies, on-the-job-training, and professional experience. All researchers were female. The interviewer did not establish a relationship with OPs prior to the study’s commencement.

Information about the interviews’ purpose and process, researchers’ roles as measure evaluators, anonymity, confidentiality conditions, and voluntary participation was given beforehand. Informed written consent was acquired prior to data collection. Additionally, OPs who consented filled in a short demographic questionnaire before or during interview conduction. Only the interviewer and OP were present during data collection. Using the interview guide, OPs were asked about facilitating and inhibiting factors for employees’ measure participation, work organization in the MHPM, personal contact with employees about RCT recruitment, inter-professional cooperation, and OPs’ overall impressions of MHPM structures and processes. OPs’ questions about researchers’ characteristics and involvement in the study (e.g., personal interest) were answered during interviews. Field notes were made in between and afterwards. The shortest interview lasted approximately 32 min, while the longest was 61 min. The mean duration was approximately 42 min. Interviews were transcribed verbatim by an external provider. Data was managed using MAXQDA 2018.

### 2.4. Conceptual Model for Data Analysis

**Figure 1:**
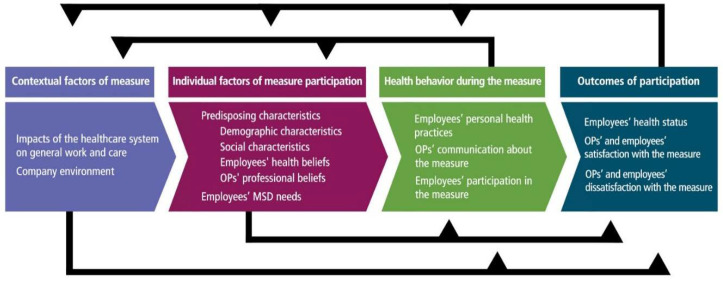
Developed categories based on Andersen’s model of health services use. OPs: Occupational Physicians; MSD: Musculoskeletal Disorders.

Andersen’s model postulates contextual factors, individual factors, health behavior, and outcomes as dimensions of health service use [13,14]. Three factors on the individual and contextual level influence health behavior and care utilization outcomes: Predisposing factors, enabling factors, and need. Predisposing factors comprise of demographics, social characteristics, and health beliefs, while enabling factors enhance or inhibit resources available for measure utilization, such as financing or care organization. Need comprises perception and professional evaluation of individuals’ functional state, risk of illness, and need for care. Health behavior comprises of individuals’ personal health practices, process of medical care, and use of personal health services. Finally, outcomes comprise of perceived health, evaluated health, and consumer satisfaction after measure utilization. The adapted categories and subcategories derived from the model in our study are presented in Figure 1.

### 2.5. Data Analysis

Three researchers, consisting of KS (rehabilitation scientist, M.A), LS, and LL, coded the data using MAXQDA 2018. Extensive peer-review and field notes were used to ensure intersubjectivity of results so as to counteract interviewer absence and possible bias in data analysis. Data was analyzed using a combination of conventional and directed content analysis [29]. Categories were first derived from the data and Andersen’s model was later used to adapt these categories and understand their relationships within a theoretical framework. Definitions of the model’s components were used as a source to map and frame discovered themes. Inductively developed categories that existed were discussed and added to the coding scheme to adapt the model’s terms based on the themes in the data. Changes to the coding scheme were repeatedly discussed and reviewed within the research team.

Initially, one interview was coded by KS, LS, and LL in teamwork to check mutual understanding of the coding scheme and overlapping of codes. Any disagreements were solved through discussion and reflection. Afterwards, three remaining interviews were coded by KS, and two remaining interviews were coded by LS and LL each. KS repeated the coding process for all materials to ensure intersubjective agreement. KEC and HP supervised data analysis and presentation of results. All coders discussed and agreed on data interpretation. Finally, quotes representative for the findings were selected, and translated from German to English. OPs had the opportunity to comment or correct transcripts, but none provided feedback.

## 3. Findings

The final sample consisted of eight interviewed OPs. Interviewed OPs’ age ranged from 41 to 62 and work experience as OPs ranged between 4 and 40 years. OPs’ general working conditions varied depending on their business location. Five OPs worked full time and five were female. Six OPs were involved in the measure since its implementation and OPs’ invested working time for the MHPM ranged between 0.5 and 4 h weekly. Six OPs concurrently served up to five business locations.

Interview guide and coding scheme are provided in Supplementary File 2. OPs reported a broad scope of factors relevant for employee participation in the measure. The analysis yielded four main categories and 10 subcategories (see Figure 1). The main categories comprised of: (a) Contextual factors of the measure, (b) individual factors of measure participation, (c) health behavior during the measure, and (d) outcomes of participation.

Data did not only comprise statements focusing the MHPM, but general contextual and individual factors outside the MHPM as well. Therefore, the categories “contextual factors” and “individual factors” rather focus on statements about general conditions relevant for MHPM participation, while the categories “health behavior” and “outcomes” focus on MHPM-specific statements. OPs did not report enabling and inhibiting factors for MHPM usage in a separate manner, but rather spoke of “scopes” and “spectrums” comprising both enabling and inhibiting factors. Thus, different from the original model [13,14], enabling resources were not added as separate categories, but were inherent in the presented categories.

### 3.1. Contextual Factors of the Measure

Two subcategories emerged in OPs’ views on contextual factors of employees’ MHPM participation. The first one addressed the impacts of the overarching national healthcare system on OPs’ work and care, while the second one addressed the companies’ established WHP structures and processes.

#### 3.1.1. Impacts of the Healthcare System on General Work and Care

Few statements about the healthcare system comprised of OPs’ duties stated by legal occupational care standards. OPs, especially those working part-time, reported a lack of time for the MHPM due to their legal duties and daily routine. They wished for better work schedule regulation in the Prevention Act and faster reactions of stakeholders like the statutory pension insurance.

*The most important thing is the Occupational Safety and Health Act, and I am already busy with simple check-ups, with specific impositions, where standards must be met, and the rest falls short. I would have liked to have much more time for it [the MHPM], but I do not get to it because I do not have the time.*

(P5)

Further statements comprised some MSD patients’ helplessness, choice overload, and negative feelings about the healthcare system. Some OPs stated that other medical professionals “put patients off” by passing the responsibility to gather medical information onto them. *They [employees] […] do not have to find out everything on their own, which is really hard in the healthcare system. […] A lot of them do not gain access to aid programs through general practitioners or specialists.* (P4)

#### 3.1.2. Company Environment

Generally, OPs reported their company’s WHP system had diverse offers. WHP structures and processes, including cooperation with health insurances and external providers, were perceived as already well established.

*The health insurance and the company itself have a very elaborate occupational health management. […] I know other companies, where there is nothing at all, and people lunge at the [MHPM] project and say: ‘Oh, this is great.’ In our company, we say: ‘Yes, this is pretty nice, too.’*

(P8)

OPs perceived interconnectedness and effective physical and communication paths between professionals as especially enabling for care processes in general. Other enabling factors comprised internal marketing for the MHPM through spreading of target-oriented information by key persons (e.g., department managers, multipliers, and health insurance), advertising material (newsletters, bulletin boards, posters, and flyers), and regular meetings with managers and employees. Employees’ access to WHP measures and MHPM facilities was considered better in rural or smaller-town settings due to low physical distances and more personal contacts. OPs perceived workers’ council as a necessary obstacle before MHPM implementation. 

### 3.2. Individual Factors of Measure Participation

Two subcategories emerged regarding OPs’ views on individual factors of employees’ MHPM participation. The first subcategory addressed predisposing characteristics comprising employees’ demographic and social characteristics, health beliefs, and OPs’ professional beliefs. The second subcategory comprised employees’ musculoskeletal health needs assessed by OPs.

#### 3.2.1. Predisposing Characteristics

Generally, OPs reported a broad scope of individual employee characteristics. They stated MHPM recruitment and adherence depended on employees’ profession, working conditions, familial ties, education, health issues, available time, motivation, and other private reasons.

Demographic characteristics: Shift structure, fluctuation, and work overload were considered problematic in terms of ageing employees with decreasing and limited working capability. MHPM participation was perceived as easier for white-collar employees, and more difficult or impossible for blue-collar employees working shifts or working internationally. Blue-collar employees in heavy physical labor were, however, seen as the MHPM’s main target group.

*I can probably get office people to do something for their health two times a week faster than people working on the assembly line for nine hours, who are really tired in the evening […]. But there are also motivated people on the assembly line who say: ‘Nah, that is important to me and that is why I invest the time.’*

(P1)

Social characteristics: Familial ties, e.g., being a young parent, were a prominent reason for employees’ inability to participate in the MHPM. OPs saw relationships between employees both positively and negatively for MHPM participation. On the one hand, OPs thought word-of-mouth did not facilitate MHPM participation appropriately, e.g., due to differing shifts, language barriers of foreign employees, and expressed criticism of measure conditions of employees. Some OPs did not see workplace settings in smaller towns fit for a RCT. *In companies, even in large companies like ours, communication is just always there among each other. This is a small village, where somehow […] everybody knows somebody, who knows somebody, who has participated in it [the MHPM]* (P1). On the other hand, participating employees informed coworkers about the MHPM, and motivated them to participate themselves.

Employees’ health beliefs: This theme comprises of OPs’ perspectives of a spectrum of employees’ WHP attitude, temperament, health behavior willingness, and employees’ WHP knowledge. OPs thought employees were generally well informed about general WHP opportunities. However, they perceived employees’ knowledge about different opportunities in MSD aid, such as out-patient measures or in-patient rehabilitation, was lacking. While most employees’ expectations regarding improvement of MSD after MHPM participation were realistic, some were too high and unrealistic, according to OPs. Towards OPs, some employees were open-minded, grateful, and happy about the opportunity to participate, while others were resistant, unimpressed, uncertain, and reluctant to engage. *Unfortunately, it is like that with all things in real life, whether you have overweight people, high blood pressure patients, smokers… If willingness does not exist, you will not seize them.* (P2)

OPs’ professional beliefs: Some OPs described a fundamental attitude and personality structure inherent in their profession. OPs saw putting effort into the MHPM as a way to live up to their own standards of staying up-to-date and innovative. One OP highlighted their own duty to give a “boost of motivation” regarding the MHPM, and to generally demand health-oriented improvements in workplace structures. Some OPs were aware of a role conflict caused by the MHPM, since they wished to provide good care as OPs, but had to refer employees to recruitment at the same time.

*There are, of course, two hearts beating within my chest, but the direction of the beat is clear. As an occupational physician, I prefer assigning someone in need of rehabilitation as soon as possible using my available options, and I do not say: ‘Great, I will put them on a stockpile so they become a perfect control group.’ […] That is how I see it. The patient is closer to me than statistics.*

(P7)

#### 3.2.2. Employees’ MSD Needs

According to OPs, there was a scope of MSD severity and needs. OPs estimated need for intervention during mandatory and optional appointments by asking about employees’ general working situation, medical and personal background. *As soon as I say the words—spine, joint problems —they latch onto it immediately.* (P5) 

If OPs considered employees “ready” and if OPs felt it “made sense” to participate, they suggested MHPM modules depending on the severity of employees’ health status, number of sickness days, and previously taken measures. Some OPs assigned employees requiring immediate intervention to other MSD measures to prevent inability to work. Assignment to the present MHPM was not reasonable to OPs if employees did not have MSD needs, if MSD complaints and needs were too severe, or if employees currently participated in a different MSD measure.

### 3.3. Health Behavior during the Measure

Health behavior comprised of three subcategories consisting of OPs’ views on employees’ personal health practices, OPs’ communication about the measure, i.e., interaction and cooperation therein, and views on employees’ participation in the MHPM.

#### 3.3.1. Employees’ Personal Health Practices

Statements about employees’ other personal health practices were few. Most frequently, OPs stated health-conscious employees, who already took care of their health in private, were more inclined towards MHPM participation. *We could have an excellent discussion whether it is how it always is, whether those, who are already health-conscious, participate in the measure.* (P7) 

Fewer employees were reported to already have experience in regular physical exercise or rehabilitation. Another frequent statement was that some employees who were not fond of assignment to control group, revisited OPs asking for an alternative MSD measure. One OP reported some employees unwilling to proactively do something for their health preferred measures like a syringe, a pill, or a massage.

#### 3.3.2. OPs’ Communication about the Measure

OPs considered actively approaching and motivating employees to participate in the MHPM to be their main contribution. Generally, OPs gave a broad informational overview of the MHPM, then referred employees to case managers “50 meters down the hallway”. Questions were mostly settled beforehand and OPs did not monitor employees continuously in the MHPM. OPs felt explaining, convincing, and motivating certain employees was challenging. Due to the study design, they could not “advertise” MHPM modules appropriately. However, most OPs reported explaining the concept in an “open and neutral” manner and did not “sugarcoat” possible control group assignment.

*When I try motivating employees to visit the case manager, I do not necessarily make their mouths water. I do not promise them heaven and earth, but explain what these modules are all about and I emphasize they run the risk of ending up in the control group.*

(P7)

If employees’ expectations about health improvements were too high, OPs communicated expected outcomes in a more realistic manner. In some cases, OPs emphasized the urgency of taking a timely MSD measure, which made some employees feel pressured. The frequency of OPs’ reported communication with case managers varied from two to three times a month. OPs reported there was little to no contact with other facilities’ OPs regarding the MHPM. 

#### 3.3.3. Employees’ Participation in the Measure

As indicated in employees’ personal health practices, OPs believed the MHPM reached those particular employees, who considered doing something for their musculoskeletal system or were already doing so. *We are pretty realistic about this. We reach those who already toyed with the idea: ‘Oh, I have to do something’. We do not reach the couch potatoes with this either.* (P8)

Admission rates were perceived as higher following MHPM implementation and decreased over time. OPs reported lower perceived case numbers in smaller facilities. Few employees in self-management were able to manage on their own and needed more intense professional guidance for adherence. However, OPs felt employees in rehabilitation needed the most support. Case number was considered very low for reintegration, i.e., for employees whose jobs were at risk due to severe MSDs. While overall drop-outs were perceived as low or non-existent, the number of employees unwilling to participate in the MHPM in the first place was considered higher than the number of willing employees.

### 3.4. Outcomes of Participation

Outcomes comprised of three subcategories consisting of OPs’ views on employees’ health status following MHPM participation, and OPs’ and employees’ satisfaction and dissatisfaction with the measure.

#### 3.4.1. Employees’ Health Status

OPs reported some employees consulting them after MHPM usage benefited in health and working performance. OPs stated those employees became attached and used to the measure, hence they maintained exercising regularly afterwards: *People often come to us and say: ‘I have been in rehabilitation, it felt good and I want to continue [...]’* (P1). However, even if health status improved, OPs perceived most employees did not continue exercising afterwards. While willingness for health behavior maintenance generally existed, OPs reported that the omitted coverage of costs induced unwillingness to maintain exercising in gym facilities. Drifting away from newly learned health-promoting behavior was perceived as the “normal course” by OPs.

*I cannot go to each machine every day and explain this to employees with weaknesses. This must get inside their heads. […] This [maintenance of behavior after rehabilitation] will be very difficult to convey because, as soon as they come home, their old habits are back within a month or two and that is the problem.*

(P5)

#### 3.4.2. OPs’ and Employees’ Satisfaction with the Measure

Overall, OPs were satisfied with the MHPM’s general conditions, such as financing, implementation, availability of information, target-orientation, internal and external cooperation, speed of administrative processes, and multi-level structure. The MHPM was considered promising, “trend-setting”. Regarding cooperation, case managers were perceived as especially skilled and helpful since they “took a lot off OPs’ hands”, reduced administration efforts, and supported employees during the measure.

Furthermore, OPs felt a bigger freedom of action since they were able to offer a more direct, specific MSD care path. Some OPs considered the MHPM a relief of choice overload patients and practitioners encounter in healthcare. *I do not have to send them around anymore and say: ‘Go to the general practitioner, go to the specialist, try some physical therapy’ or something, but I can simply suggest a promising, viable way. That has changed and I find that very, very helpful* (P4). Additionally, OPs considered the MHPM an opportunity to facilitate awareness of MSDs and WHP in the company.

According to OPs, employees generally reported being satisfied, especially in treatment groups, early intervention, and rehabilitation. OPs perceived that employees liked the closeness of facilities, and the custom-tailored character of the measure. *They [employees in self-management] enjoy someone attentively looking after them now and offering them something. This is mostly positive* (P2). 

#### 3.4.3. OPs’ and Employees’ Dissatisfaction with the Measure

OPs reported disappointment, dissatisfaction, and sadness about low case numbers, employees’ lacking maintenance of learned behavior, and stagnating enthusiasm about the MHPM in the company. They criticized the reintegration module for its negative cost-benefit ratio, and quality of rehabilitation follow-up care. Existence of control groups, especially in the reintegration module, hindered OPs in assigning employees. *This module is about whether you need to assign someone to another workplace. That is where I see the problem... what do we do with a person who enters the placebo group? I cannot leave them out in the rain* (P3). Some OPs felt the MHPM was “a step backwards” for the improvement of employee healthcare. Additionally, OPs reported uncertainty about the final “conclusion” of the MHPM. Furthermore, OPs wished for more and earlier feedback on employees’ progress to be able to advertise it better. Most OPs reported they only gained knowledge about that by meeting an employee “coincidentally” later.

OPs reported employees were dissatisfied because the MHPM was time-consuming and success did not come quickly. Across all modules, potential and actual control group assignment was perceived as frustrating, demotivating, and disappointing. *They [employees] say things like: ‘I went through all this stuff and now I am supposed to wait a year?’ […] If it [control group] befalls them, they do not find it that great. That is understandable* (P7).

## 4. Discussion

In this qualitative study, we investigated OPs’ views on employee participation in a RCT of a musculoskeletal measure at the workplace, contextual and individual factors, and reported outcomes. Findings are discussed considering previous quantitative and qualitative research.

By mapping categories to Andersen’s model, we identified employee-, occupational physician-, and organization-related determinants. Unlike other models, such as the Theory of Planned Behavior [34], the Transtheoretical Model [35], or the Health Belief Model [36], Andersen’s model suited the explanation of our findings since its multilevel structure allows the incorporation of both individual and contextual determinants of WHP participation [15]. However, findings need to be considered in light of the bias this deductive approach implicates, and with sufficient skepticism due to the perspectives of OPs and researchers. Since the measure is embedded within a RCT, group assignment and RCT context need to be considered regarding the conclusions of this study. 

Factors of WHP participation mentioned by OPs were complementary to factors mentioned by employees [25] or managers [26] in qualitative studies. Regarding participation in WHP measures, time and financial constraints were mentioned as major barriers by employees before [24]. Our findings underline that time and omitted financial incentives are impeding factors for participation and maintenance of health-promoting behavior from OPs’ perspectives. Furthermore, a low sense of control, lack of self-efficacy, and negative cost-benefit ratio were mentioned by employees’ as reasons for non-adherent behavior before [25]. Employees’ uncertainty about group assignment in the present RCT and some employees’ negative feelings about the healthcare system support the finding that a lack of perceived control and self-efficacy affect measure participation negatively [37]. However, findings of the category “employees’ health status” imply experiencing improvement of MSD complaints may facilitate self-efficacy and maintenance of health-promoting behavior. Managers’ perceptions of employees’ WHP uptake in another qualitative study comprised awareness of WHP, and attitudes as individual factors [26]. The existence of a scope of employees’ differing attitudes facilitating or impeding participation correspond with our findings of OPs’ views. OPs in our study especially emphasized the role of previous health-promoting behavior for measure participation. Regarding awareness, some OPs perceived employees are sufficiently informed about WHP, while others perceived they are not. This rather supports findings that employees’ knowledge about WHP and MSD measures needs to be facilitated on a broader scale [6]. The scope of individual characteristics reported by OPs further supports researchers’ demand for target-orientation and inclusion of employees in decision-making processes in WHP [38,39].

In a different quantitative study, organizational determinants of employees’ WHP participation were focused on: Strong organizational support had a positive impact, while employees’ co-payment and firm size had a negative impact on participation [40]. In line with other findings, OPs’ statements in our study suggest social environment and support have an essential impact on employees’ perception of a measure, e.g., through encouraging or skeptical communication of colleagues or contact persons [22,26]. While physician-patient relationship did not emerge as a major theme in our study, findings on OPs’ communication with employees suggest the significance of their interaction for employees’ perceptions of health needs [21]. OPs’ statements further implicate inter-professional communication with OPs, e.g., with rehabilitation professionals or case managers, remains insufficient in Germany [12,41,42]. Disparities in perceptions of cooperation in the present RCT underline the meaning of practical development of shared goals and cooperation in occupational healthcare settings [27,43].

Regarding firm size, OPs’ disappointed expectations about admission numbers indicate that further measures are needed to increase participation in larger companies. While OPs were mostly satisfied with general organizational conditions regarding the MHPM, OPs’ criticism in our study also suggests organizational conditions such as their involvement in inter-professional communication or their working conditions need to be improved for better WHP quality. Challenges reported by managers in executing WHP in another qualitative study were mostly at the organizational level as well [26]. This underlines the meaning of organizational influences on measure participation and change therein. 

Lastly, few studies have investigated participant recruitment challenges in occupational health care [37]. Regarding the present RCT study design, recruitment and lacking enrollment were identified as a common problem for multicenter RCTs before [44]. Case managers as main responsible agents for MHPM coordination reported similar challenges as OPs due to group assignment and group terms [27]. Researchers should therefore consider WHP-specific contexts, OPs’ roles, and employees’ negative perceptions of control groups for participation in future study designs. Researchers may also use knowledge e.g., from findings of categories “company environment”, “OPs’ communication about the measure”, and “OPs’ and employees (dis-)satisfaction” to better understand how to engage OPs in the recruitment of research participants. 

## 5. Strengths and Limitations

This study’s strengths comprise of the application of Andersen’s model to a WHP context and OPs’ views on employee participation, where research is sparse. To the best of our knowledge, Andersen’s model has not been adopted for qualitative analysis of OPs’ perspectives in the context of WHP yet. Thus, present findings have value regarding their explorative character. Study limitations comprise relatively low sample size, data saturation, lacking generalizability, and transferability of findings to routine WHP. Nonetheless, basic elements for meta-themes in the data can arise as early as six interviews [45]. While implications are limited to the context of the RCT design, diverse working conditions among OPs, e.g., different business locations, constitute a strength regarding transferability of findings. This is underlined by OPs reporting about WHP and employee participation on a general level, independent of the underlying study design. While this study was conducted in Germany, there are aspects of this study that can be useful regarding OPs and WHP worldwide. Yet, this study illuminated solely OPs’ perspectives. Purposive sampling was determined by OPs’ involvement in the MHPM and may have caused selection bias. Hence, socially desirable answers due to OPs’ employment in companies and strong interest in the topic could not be controlled. However, interviewing OPs also constitutes a study strength since employees might have been more socially biased in answers had they been asked directly. Additionally, recruiting physicians for health services research is challenging due to their lack of time and capacity [46,47,48]. Thus, the difficulty of accessing a sample of OPs adds further value to the study.

Higher data saturation can be achieved in future studies, preferably using face-to-face interviews, as these provide further important observational and contextual information. Due to one-time data collection, change of OPs’ perceptions over time was not explored. Results of this study could, however, be compared to findings of repeat interviews.

## 6. Conclusions

This explorative, qualitative study illustrates OPs’ perceptions about a broad range of individual and contextual factors for employees’ participation in a RCT of a musculoskeletal health promotion measure. The study complements the research on Andersen’s model, underlining that WHP and RCT participation is a multilayered phenomenon that is affected by employee-, physician- and organization-related determinants.

Further studies can apply sample selection criteria for OPs based on this study and investigate the topic on a broader scale, and consider OPs’ statements for the development of intervention study designs at workplace settings. Researchers may use category findings to account for barriers and facilitators for participant recruitment on a physician and organizational level when developing RCTs. Additionally, OPs, occupational health care professionals, and employees can use findings to better understand their behavior, each other, and their organization.

The findings implied there is potential for improvement in legal and organizational matters in prevention of MSDs and WHP, e.g., regarding the execution of the Prevention Act, inter-professional communication, OPs’ working conditions, or target-orientation in WHP measures. Stakeholders such as company managers, authorities in social security organizations, or policy makers can use findings for the development of WHP programs that address these issues. However, further studies should investigate other stakeholders’ and employees’ perspectives on WHP participation to probe these implications.

## Supplementary Materials

The following are available online at https://www.mdpi.com/1660-4601/17/20/7445/s1: description of RCT modules and program (supplementary file 1), interview guide, and coding scheme (supplementary file 2).

## Abbreviations

WHP	Workplace Health Promotion
MSDs	Musculoskeletal Disorders
OPs	Occupational Physicians
RCT	Randomized Controlled Trial
MHPM	Musculoskeletal Health Promotion Measure
