# Supplementary material for: Occupational Physicians’ Perspectives on Determinants of Employee Participation in a Randomized Controlled Musculoskeletal Health Promotion Measure: A Qualitative Study
Source: Int J Environ Res Public Health. 2020 Oct 13;17(20):7445. doi: 10.3390/ijerph17207445 (PMC7650758; doi:10.3390/ijerph17207445)
Supplement: Supplementary file 1 [file ijerph-17-07445-s001.zip › Suppl file 1_module and program description.docx]

Description of modules and MHPM program

**Background on the program and implementation:** The measure described in the article offers individual and workplace-related healthcare for employees with musculoskeletal disorders (MSD). Its main innovation is the development of an extensive healthcare network, as care processes are rarely connected and coordinated among German social insurance institutions. The randomized controlled trial (RCT) aims at the evaluation of the new program’s (MHPM’s) effectiveness. In order to use the existent organizational systems in place as efficiently as possible, professional duties within the measure were assigned to stakeholders in the existent health-promoting network instead of outsourcing the tasks to disconnected study staff. This is supposed to facilitate possible implementation on a larger scale in businesses after positive evaluation.

**Occupational physicians’ (OPs’) role:** As mentioned in the article, OPs are responsible for taking the medical history of eligible employees, checking their inclusion and exclusion criteria, informing them about modules and assist recruitment by referring to case managers with a recommendation for a specific module. OPs received a two-day training and instruction manuals explaining the measure’s multimodal structure and study design prior to implementation. In the manual, OPs find further information on the program and their specific tasks within (e.g. on modules, recruiting, describing employees’ workplace, writing medical reports, or making appointments for follow-up care).

**Early intervention (Module A)**: This module aims at minor MSD complaints and few sick leave days. In self-management of early intervention, case managers give employees tailored information about regular MSD healthcare measures for light complaints, and a thera-band with an instruction manual, so employees can exercise on their own. In case management of early intervention, employees receive a workplace related diagnostic test and a tailored 13-week training program for muscle build-up in a gym.

**Rehabilitation (Module B)**: This module aims at moderate complaints and mid-range sick leave days. In self-management of rehabilitation, case managers give employees tailored information about regular MSD healthcare measures for distinctive complaints and the option of applying for in-patient or out-patient workplace related rehabilitation on their own. In case management of rehabilitation, employees participate in a workplace-related in-patient or out-patient rehabilitation, that case managers filed and coordinated.

**Reintegration (Module C)**: This module aims at major complaints, increased sick leave days, and limited working capability. In self-management of reintegration, case managers give employees tailored information about regular MSD healthcare measures for severe complaints. In case management of reintegration, employees receive detailed workplace-related diagnostic test and a psychological assessment for employability. Based on these tests and assessments, further measures (e.g. in-patient or out-patient rehabilitation in Module B, gradual reintegration into the workplace, aid for participation at work) are assigned to employees.

**Case management (treatment group):** Different from standard healthcare in Germany, the insured persons in the treatment group are additionally given thorough information about possible support offers in the respective modules. Most importantly, case managers accompany the trial process and help participants e.g. with making appointments, provide feedback on training and motivate participants. They serve as contact persons and have an overview of the entire process. If necessary, they can mediate between involved stakeholders.

**Self-management (control group):** This term refers to the control group receiving standard care or "treatment as usual". In the self-management group, participants are not instructed or thoroughly supported by case managers, and manage the training themselves. However, they can complete a muscular training or apply for rehabilitation, independent of participation in the trial.
