# Supplementary material for: Occupational Physicians’ Perspectives on Determinants of Employee Participation in a Randomized Controlled Musculoskeletal Health Promotion Measure: A Qualitative Study
Source: Int J Environ Res Public Health. 2020 Oct 13;17(20):7445. doi: 10.3390/ijerph17207445 (PMC7650758; doi:10.3390/ijerph17207445)
Supplement: Supplementary file 1 [file ijerph-17-07445-s001.zip › Suppl file 2_Interview guide and coding scheme_revised.docx]

Semi-structured interview guide for occupational physicians’ experiences with a musculoskeletal health promotion measure

| **Main question** | **Specified question** | **Key words** |
| --- | --- | --- |
| **1. Beginning** | | |
| ***Introduction*** | *Introduction of interviewer*  *Clarification of interview aims*  *Gratitude for interviewee’s time* |  |
|  | *You can answer the questions openly and in detail. Your data will be anonymized.* |  |
| Entry question:  Did you already talk to employees or supervise them about the project? | If not, why so? |  |
| How do you identify employees for the project? | How do you proceed when you contact a potential candidate?  Do you approach them directly?  Do you take the initiative or the case manager? | Communication between colleagues about employees |
|  |  |  |
| **Main question** | **Specified question** | **Key words** |
| **2. Main part** | | |
| **a) Key word: Occupational physicians‘ (OP) work organization** | | |
| Please describe the procedure of counselling your employees. How does your work change due to the project regarding organization and procedure? | What does OP supervision look like in general?  What was it like before the project?  How does participation in a study affect every day work requirements?  Which aspects of the project do you consider positive for employees, the company and your own way of working?  Which aspects are considered negative? | Process of supervision  Process of counselling  Change in organization |
| Would you say your way of counselling employees changes when they are in the study? | If so, how would you describe the change in counselling?  How do you feel about being able to offer employees measures in both case management and self-management?  Do you talk to them differently if you consider them for assignment? | Change in counselling and supervision |
| **b) Key word: Contact with employees and counselling** | | |
| How much time do you take for the project? Are there differences between the modules (B and C)? | How do you feel about that?  How does referral to the case manager work?  Can you tell me about a case that took more time than you thought? | Time for employee  Patient care |
| How do employees react to the option of participating in the study when you suggest it? What do employees say about their expectations? | What hopes do employees have about their group assignment and recuperation?  Would you say reactions differ depending on assignment to case management or self-management?  If so, could you describe the reactions in more detail? | Reaction and interest of employees towards measure participation |
| **c) Key word: Personal experiences** |  |  |
| What kind of experiences did you already gather in the project? | What is going well within the project? What is going poorly?  What is your attitude towards studies in general?  Do your experiences in this project differ from previous experiences in other studies or projects? | Gained insights due to project |
| Would you say you feel well informed about the project in general? | 1. How did spreading of information about the project work in your company?  In what form did you receive information? Who informed you? Was information sufficient? What information material is available to you?  2. How is this for employees? Is there sufficient information material for employees?  How does an employee find out who they have to contact if they want to participate in the study? | Conditions at the start of the project and knowledge about the project |
| Please describe your inclusion in the project through other colleagues (other OPs, project leaders, and case managers). | How do you feel about communication with other colleagues (other OPs, project leaders, and case managers)? Can you give me an example?  How frequently do you communicate with them?  How does cooperation work? Please describe cooperation with other colleagues.  Are there any problems? | Inclusion in the project  Exchange with colleagues |

| **Main question** | **Specified question** | **Key words** |
| --- | --- | --- |
| **3. End and gratitude for interview** | | |
| Do you have suggestions or requests regarding your work in the project? | Do you need more time, contact with others, information etc.?  Where do you see room for improvement in the study? | Wishes towards cooperation with other colleagues  Improvements regarding study |
| Do you have requests regarding your inclusion in the project, e.g. regarding cooperation with other colleagues? | Do you want to comment on contact with others or exchange of information? | Wishes towards inclusion in the project |
| Would you like to add something yourself? |  |  |

Coding scheme of developed categories and subcategories based on Andersen’s model of health services use

| **Category** | **Subcategory** | **Definition** |
| --- | --- | --- |
| **a) Contextual factors** **of the measure** | **a.1) Impacts of the healthcare system on general work and care** | Healthcare system comprises statements regarding beliefs, convictions, emotions or perceived facts about the national healthcare system. |
|  | **a.2) Company environment** | Company environment comprises descriptions of established structures and processes in the local company relevant for the general workplace health promotion (WHP) system, and pathways between cooperating professionals (e.g. physical, administrative paths). Statements include factors for implementation and usage of the musculoskeletal health promotion measure (MHPM) that OPs consider enabling or inhibiting. |
| **b) Individual factors** **of measure participation** | **b.1) Predisposing characteristics** | Predisposing characteristics include statements about employees' demographic characteristics, social characteristics and health beliefs, which can be stated as enabling or inhibiting for MHPM usage. They also include the inductively developed subcategory "OPs' professional beliefs". |
|  | **b.1.1) Demographic characteristics** | Demographic characteristics comprise employee characteristics, such as gender, age, education or reported profession. |
|  | **b.1.2) Social characteristics** | Social characteristics comprise employee characteristics, such as family ties, relationships with coworkers, and communal factors. |
|  | **b.1.3.1) Employees' health beliefs** | Health beliefs comprise employees' cognitions towards health and the MHPM, such as attitude, motivation, knowledge, expectations, or emotions prior to MHPM participation. This category excludes the subcategories "satisfaction" and "dissatisfaction" following MHPM assignment and usage. |
|  | **b.1.3.2) OPs' professional beliefs** | Professional beliefs comprise OPs' cognitions towards their own professional role inside and outside the MHPM, such as attitude, emotions or role conflicts. This category excludes the subcategories "satisfaction" and "dissatisfaction" following employees' MHPM assignment and usage. |
|  | **b.2) Employees’ MSD needs** | Need consists of employees' reported musculoskeletal health needs and assessed musculoskeletal health needs by OPs. This category includes statements about diagnostic processes regarding need assessment specifically, which is excluded in the subcategory "care process". |
| **c) Health behavior** **during the measure** | **c.1) Employees’ personal health practices** | Personal health practices include employee practices not associated with the MHPM, such as other WHP measures or private measures. |
|  | **c.2) OPs’ communication about the measure** | Care process comprises OPs' statements about personal interactions in the delivery of MHPM-relevant care, e.g. the quality of OP-employee communication about MHPM recruitment, or general patient counselling. Interactions include OPs' communication with other professionals, e.g. with case managers regarding employee recruitment. |
|  | **c.3) Employees’ participation in the measure** | Measure usage comprises statements about perceived reach of employees, admission rates, drop-out rates, and adherence in the MHPM. |
| **d) Outcomes of participation** | **d.1) Employees’ health status** | Health status includes perceived improvement of health and capability due to the MHPM, and following maintenance of health promoting behavior. This category excludes the subcategory "satisfaction" with overall measure conditions. |
|  | **d.2) OPs’ and employees’ satisfaction with the measure** | Satisfaction comprises CPs' and employees' reported satisfaction with the MHPM following measure assignment and usage. The subcategory covers satisfaction with MHPM aspects such as convenience, availability, financing, provider characteristics and quality. |
|  | **d.3) OPs’ and employees’ dissatisfaction with the measure** | Dissatisfaction comprises CPs' and employees' reported dissatisfaction with the MHPM following measure assignment and usage. The subcategory covers dissatisfaction with MHPM aspects such as convenience, availability, financing, provider characteristics and quality. The category also comprises wishes and suggestions for future MHPM improvement. |
